# O_2_ permeability of lipid bilayers is low, but increases with membrane cholesterol

**DOI:** 10.1007/s00018-021-03974-9

**Published:** 2021-10-25

**Authors:** Samer Al-Samir, Fabian Itel, Jan Hegermann, Gerolf Gros, Georgios Tsiavaliaris, Volker Endeward

**Affiliations:** 1 AG Vegetative Physiologie 4220, Zentrum Physiologie, Medizinische Hochschule Hannover, 30625 Hannover, Germany; 2 Empa, Swiss Federal Laboratories for Materials Science and Technology, Lerchenfeldstr. 5, CH-9014 St. Gallen, Switzerland; 3 Abteilung Funktionelle und Angewandte Anatomie, Elektronenmikroskopie 8840, Medizinische Hochschule Hannover, 30625 Hannover, Germany; 4 Abteilung Biophysikalische Chemie 4350, Medizinische Hochschule Hannover, 30625 Hannover, Germany

**Keywords:** Hemoglobin deoxygenation, Hemoglobin-loaded liposomes, O_2_ net flux across membranes, Stopped-flow technique, Deoxygenation by mixing with dithionite

## Abstract

Oxygen on its transport route from lung to tissue mitochondria has to cross several cell membranes. The permeability value of membranes for O_2_ (P_O2_), although of fundamental importance, is controversial. Previous studies by mostly indirect methods diverge between 0.6 and 125 cm/s. Here, we use a most direct approach by observing transmembrane O_2_ fluxes out of 100 nm liposomes at defined transmembrane O_2_ gradients in a stopped-flow system. Due to the small size of the liposomes intra- as well as extraliposomal diffusion processes do not affect the overall kinetics of the O_2_ release process. We find, for cholesterol-free liposomes, the unexpectedly low P_O2_ value of 0.03 cm/s at 35 °C. This P_O2_ would present a serious obstacle to O_2_ entering or leaving the erythrocyte. Cholesterol turns out to be a novel major modifier of P_O2_, able to increase P_O2_ by an order of magnitude. With a membrane cholesterol of 45 mol% as it occurs in erythrocytes, P_O2_ rises to 0.2 cm/s at 35 °C. This P_O2_ is just sufficient to ensure complete O_2_ loading during passage of erythrocytes through the lung’s capillary bed under the conditions of rest as well as maximal exercise.

## Introduction

It has long been thought that the respiratory gases permeate all cell membranes without noticeable resistance. In recent years, however, this assumption has been rebutted by several studies, at least for carbon dioxide, for which it has been shown that membranes can present a significant diffusion resistance that drastically rises with higher cholesterol levels in the membrane [1–6], and on the other hand, can be greatly alleviated by the incorporation of protein gas channels such as aquaporin-1 or Rhesus-associated glycoprotein [2, 7–10]. In the case of the permeation of oxygen across membranes, current data mainly obtained from ESR spin label studies [11], or other indirect biophysical techniques such as fluorescence quenching and molecular dynamics (MD) simulations [12–14] have reported for liposomes extremely high permeabilities of 20–125 cm/s that would physiologically amount to zero resistance in the case of a cell membrane. O_2_ permeabilities between 10 and 100 cm/s are shown at the end of this paper to exert no detectable influence on the deoxygenation kinetics of red cells. Thus, such P_O2_ values have no influence on the overall deoxygenation process, whose kinetics then is determined by the hemoglobin deoxygenation kinetics and by intracellular diffusion only.

So far, no O_2_ permeabilities of liposomes have been determined from direct measurements of O_2_ flux across the liposome membrane. The only reasonably sensitive P_O2_ determination based on an O_2_ flux observed across a cell membrane to our knowledge is that of Holland et al. [15], who report an orders of magnitude lower P_O2_ value of 0.6–0.8 cm/s for the red cell membrane using the stopped-flow technique. This discrepancy prompted us to readdress the question of membrane permeability to O_2_, following the strategy of using—instead of cells—the much simpler model of unilamellar lipid vesicles of 50 nm radius loaded with oxyhemoglobin. We measured the efflux of O_2_ across the lipid membrane of this artificial but easily modifiable system. This was achieved by mixing the suspension of oxyhemoglobin-loaded liposomes in a rapid reaction stopped-flow apparatus with a 50 mM dithionite solution that within 1 ms consumes dissolved O_2_ in the extraliposomal space, and thus practically instantaneously after mixing has established a zero partial pressure of O_2_ in this space. The O_2_ diffusing out of the liposome after dissociation from hemoglobin was quantitated by spectrophotometry of the intraliposomal hemoglobin at a suitable wavelength and followed over time. The time course of the observed transients represents the deoxygenation kinetics of the intraliposomal oxyhemoglobin. To derive the P_O2_ of the liposome membrane, we compared this kinetics with that of pure hemoglobin solution. We prepared liposomes from the most common lipids found in mammalian cell membranes, namely phosphatidylcholine (PC), phosphatidylethanolamine (PE) at a ratio of 8:2 (moles/mole) and different cholesterol (Chol) concentrations ranging from 0 up to 70 mol%.

The major physiological message from this study is that phospholipid membranes containing cholesterol in a concentration equalling that of the erythrocyte membrane (45 mol%), possess a P_O2_ not far in excess of physiological requirements but well adapted to physiological needs, and just sufficient to allow complete blood oxygenation in the lung at rest as well as at maximal aerobic exercise.

## Results

### Properties of liposomes

**Fig. 1:**
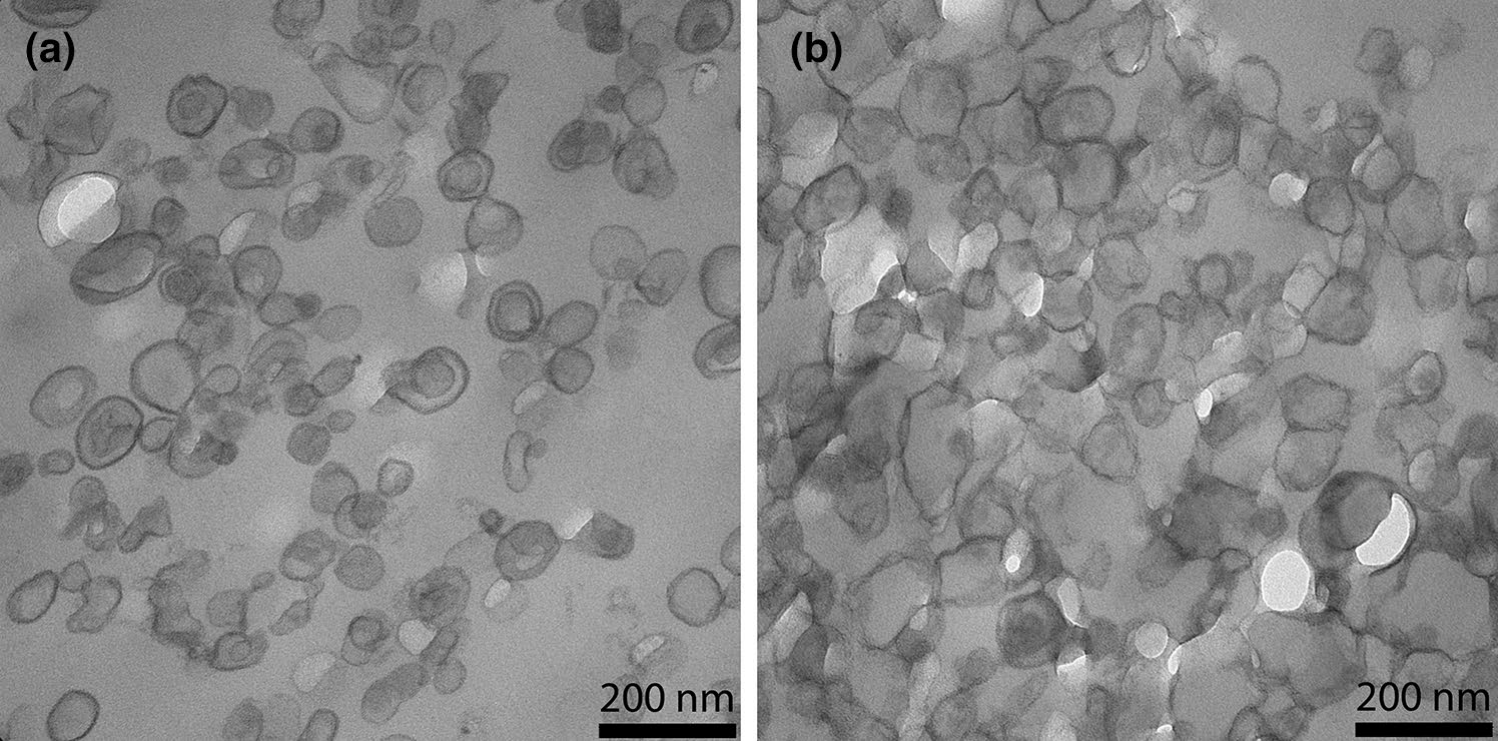
Transmission electron micrographs of liposomes containing 0 (**a**) and 50 mol% cholesterol (**b**) in their membrane

**Fig. 2:**
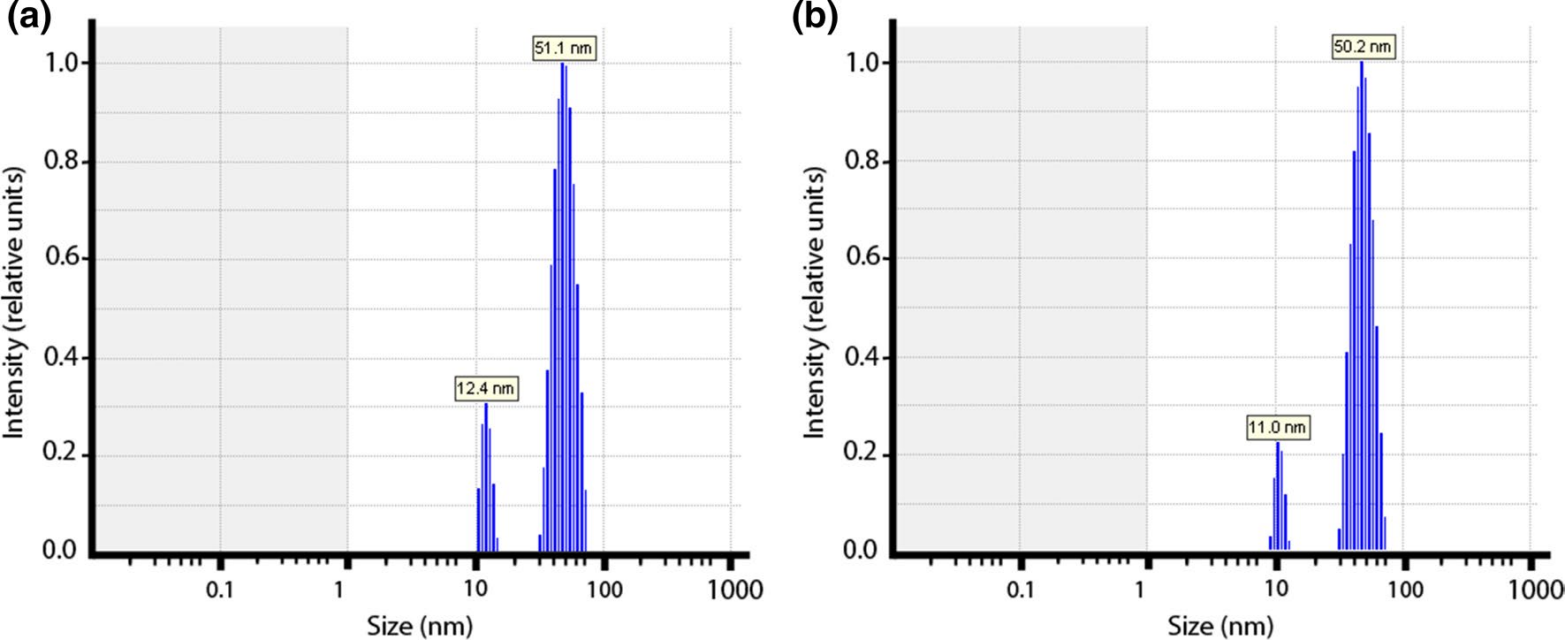
Vesicle size distributions by dynamic light scattering. **a** Liposomes without membrane cholesterol exhibit an average radius of 51.1 nm, **b** liposomes with 50 mol% cholesterol an average radius of 50.2 nm. All liposome preparations gave similar distributions with radii of around 50 nm. There were only minor contaminations with particles of a radius around 10 nm

Here, we studied aspects of the liposomes that are important for the interpretation of the observed O_2_ effluxes in terms of liposomal membrane O_2_ permeability: are the liposomes unilamellar or multilamellar? What is the size and size distribution of the liposomes? How well are higher amounts of cholesterol incorporated into the membrane? Fig. 1 shows electron micrographs of liposomes with cholesterol contents of 0 and 50 mol%. It is apparent that for both types of vesicles the diameters are around 100 nm. In the presence of cholesterol the liposomes are entirely unilamellar, only in the complete absence of cholesterol a minor fraction has two membranes, an outer and an inner one. This fraction was quantified as constituting 12%. To quantitate the size of the liposomes, we used dynamic light scattering, DLS, providing the intensity size distributions exemplified in Fig. 2. Figure 2a and b show both narrow size distributions with average radii of the liposomes of about 50 nm [a: 51.1 ± 11 (SD) nm, b: 50.2 ± 8.2 (SD) nm]. This radius was found with minor variations (50–57 nm) in all the preparations used here. Figure 2 shows that contamination by lower size particles was very minor. A radius of 50 nm is in excellent agreement with the liposome diameter of around 100 nm as apparent in the electron micrographs of Fig. 1*.* To validate the incorporation of cholesterol into the lipid bilayer, we experimentally quantified the cholesterol and phosphatidylcholine content of the liposomes. Between 0 and 50 mol% cholesterol, the experimental ratios of cholesterol/phosphatidylcholine closely follow the “theoretical” values as they are present in the initial lipid mixture from which the liposomes are generated. Only at cholesterol concentrations above 50 mol% the experimental ratios are somewhat lower than the theoretical ratios. This suggests that cholesterol incorporation up to 50 mol% is proportional to the cholesterol concentration present in the lipid mixture, while above 50 mol% cholesterol incorporation into the lipid bilayer begins to become limited. It should be noted that in the results given below, we always use the cholesterol fractions expected from the lipid composition employed for preparation (theoretical value).

### Kinetics of the deoxygenation of oxyhemoglobin-loaded liposomes

**Fig. 3:**
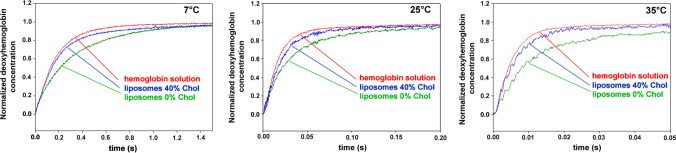
Original records of stopped-flow experiments with hemoglobin solutions and liposome suspensions. *Y*-axis: the normalized relative change of absorbance at 436 nm (liposomes) or 470 nm (hemoglobin solutions), respectively. The records show the disappearance of oxyhemoglobin after mixing with dithionite solution. Red (and uppermost) curves hemoglobin solution, blue (and middle) curves liposomes with 40 mol% cholesterol, green (and lowermost) curves liposomes with 0% cholesterol. At the three temperatures 7 °, 25 ° and 35 °C, the kinetics of all liposomes is slower than that of hemoglobin solution, the liposomes with 0 mol% cholesterol exhibiting the slowest kinetics. All records represent the averages of 8 single stopped-flow shots. Normalization of the *Y*-axis was done by subtracting the starting absorbance value from the curve and setting the amplitude of the signal to 1.0

**Fig. 4:**
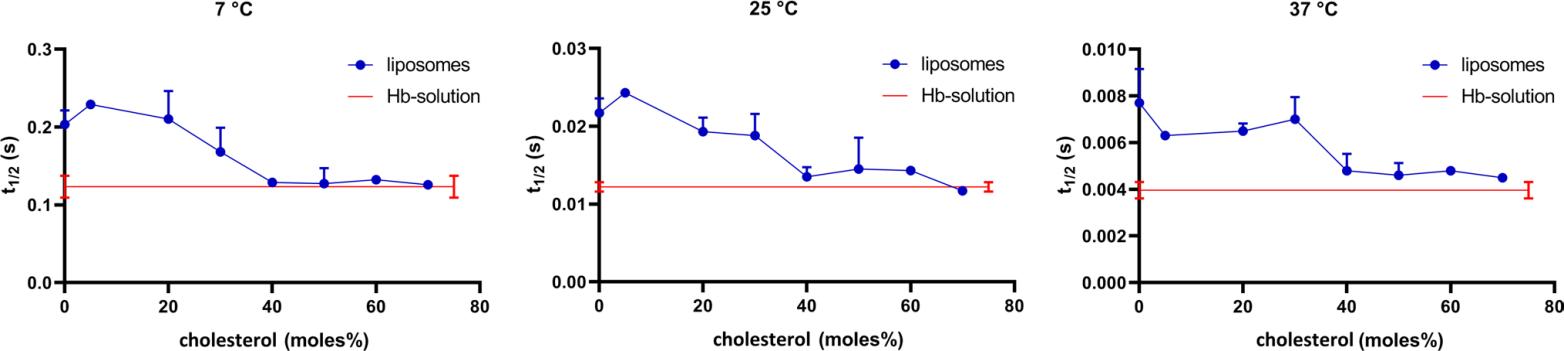
Half-times t_1/2_ of the stopped-flow deoxygenation kinetics of hemoglobin-loaded liposomes and pure hemoglobin solutions. Membrane cholesterol clearly reduces the difference between liposomes and pure hemoglobin solutions. At all temperatures, most of this effect occurs between 20 or 30 and 40 mol% cholesterol. Each point represents between 2 and 4 experimental stopped-flow series, each series comprising 8 single stopped-flow shots. n for hemoglobin solutions was ≥ 8. Bars represent S.D. values where applicable

Here, we present the results of stopped-flow experiments measuring the kinetics of hemoglobin deoxygenation and O_2_ efflux from liposomes. Figure 3 shows original stopped-flow tracings at the three temperatures studied for pure hemoglobin solution, and for suspensions of liposomes loaded with oxyhemoglobin with 0 and 40 mol% membrane cholesterol (other cholesterol concentrations not shown). All the liposomes possess a slower kinetics than hemoglobin solution. However, the difference is greatest for the liposomes lacking cholesterol in their membrane, and smallest for the liposomes having 40 mol% cholesterol. Thus, it appears that the presence of a membrane between the hemoglobin and the dithionite slows down the deoxygenation process to an extent dependent on membrane cholesterol. The kinetics of the original stopped-flow tracings are quantified in terms of half-times in Fig. 4, which shows the results for all liposomal cholesterol concentrations studied. The curves confirm that liposomes with 0% cholesterol exhibit the slowest kinetics, while with 40% cholesterol an almost twice as fast kinetics is observed, and the half-times approach the values of the hemoglobin solutions. The results of Fig. 4 can be summarized in three points: (1) between a membrane cholesterol of 0 and 20 mol%, the half-times of the deoxygenation kinetics are rather similar to each other and are all about 2 × greater than those of pure hemoglobin; (2) between membrane cholesterol concentrations of 40 and 70 mol%, half-times are also quite similar at a considerably lower level, being only slightly above those of pure hemoglobin solution; (3) between 30 and 40 mol% cholesterol, a step seems to occur in all curves that is associated with a drastic decrease in half-times. This suggests that between 30 and 40% the slowing influence of the membrane on t_1/2_ diminishes greatly.

**Fig. 5:**
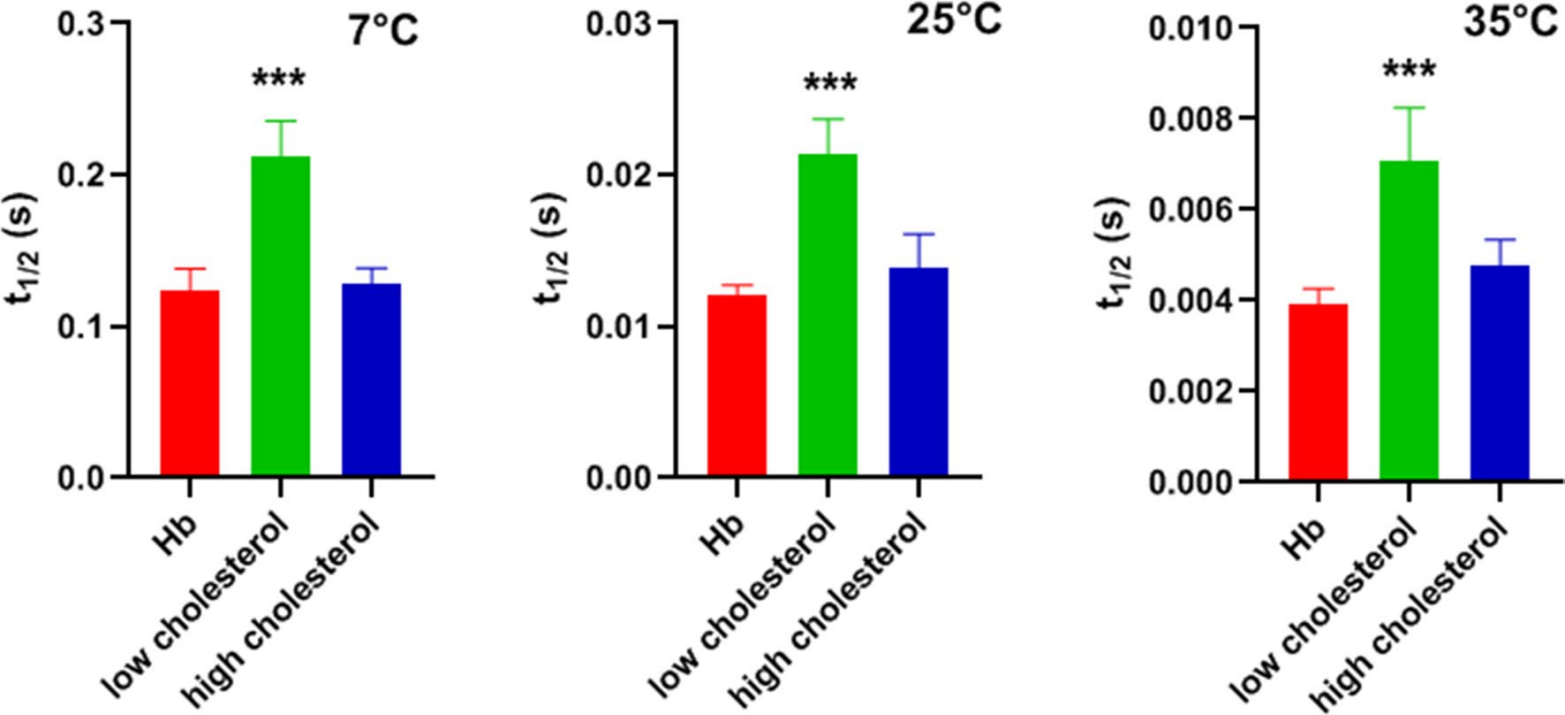
Half-times for hemoglobin solutions, Hb, and low- and high-cholesterol liposomes, respectively. “Low cholesterol” represents all data of Fig. 4 between 0 and 20 mol% cholesterol, “high cholesterol” comprises the data of Fig. 4 for 40–60 mol%. One-Way ANOVA with Tukey post-test shows that low cholesterol t_1/2_ is significantly greater than hemoglobin t_1/2_ at all temperatures. “High cholesterol” data are also somewhat greater than hemoglobin t_1/2_, but the difference does not reach statistical significance. Number of t_1/2_ values per column is 9 for Hb, 6 for low cholesterol, and 6 for high cholesterol. ***Indicates *p *< 0.0001

Figure 5 shows that the low-cholesterol t_1/2_ values are highly statistically significantly different from the t_1/2_ values for hemoglobin solution (*p* < 0.0001 at all temperatures). “Low-cholesterol” was defined here as all t_1/2_ values between 0 and 20 mol% cholesterol, a combination, which seems justified in view of the fact that there are only minor differences within this group. “High cholesterol” was defined as all values between 40 and 60 mol%, which again show hardly any differences within themselves. As “high-cholesterol” is fairly close to the hemoglobin values, it is not surprising that the difference between these two groups is not statistically significant (*p* = 0.84, 0.12, 0.096 at 7, 25 and 35 °C, respectively).

**Fig. 6:**
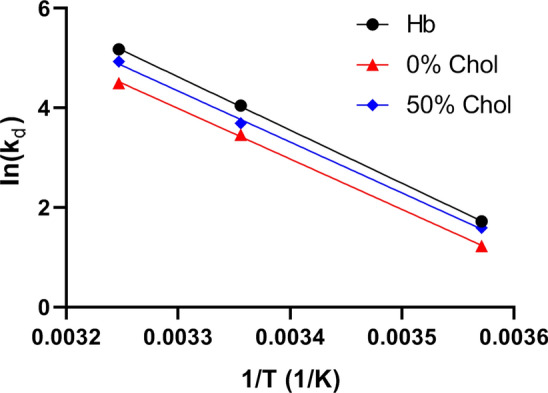
Arrhenius plot describing the temperature dependence of the dissociation kinetics of O_2_ from hemoglobin, in solution and within liposomes. *k*_*d*_ represents the apparent experimental dissociation reaction constant of HbO_2_ (T is temperature in Kelvin). The activation energy Ea for all three solutions/suspensions is calculated to be 90 kJ/mol

The temperature dependence of the kinetics of deoxygenation is illustrated in the Arrhenius plot shown in Fig. 6. The *Y*-axis represents the apparent dissociation constant *k*_*d*_ and was derived from the experimental t_1/2_ of Fig. 4 as *k*_*d*_ = ln2/t_1/2_. The slope in Fig. 6 is about identical for hemoglobin solution, 0% Chol liposomes and 50% Chol liposomes. Thus, the activation energy is about 90 kJ/mol for all three systems. This is in fair agreement with activation energies previously reported for the oxyhemoglobin dissociation reaction [16]. This illustrates that the activation energy of the chemical reaction dominates also the combined reaction–diffusion process in liposomes. The intercept on the *Y*-axis represents ln (A), where A is the pre-exponential factor of the Arrhenius equation. ln (A) in Fig. 6 decreases in a systematic fashion with increasing diffusion resistance of the liposome membrane.

### Oxygen permeability of liposomes

**Fig. 7:**
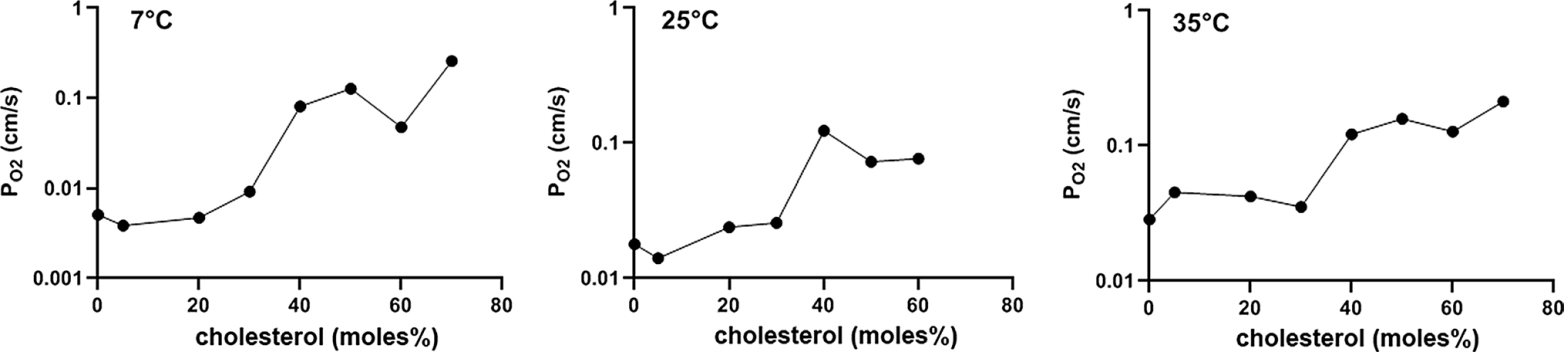
Oxygen permeabilities of liposomes at 7, 25 and 35 °C. All permeabilities are lowest in the absence of cholesterol, and increase by about one order of magnitude with increasing cholesterol content in the membrane

On the basis of the theory of O_2_ release by liposomes described in “Methods”, we have derived P_O2_ values (Fig. 7) from the experimental t_1/2_ values given in Fig. 4. The O_2_ permeabilities in the absence of cholesterol are 0.0051 cm/s, 0.018 cm/s, and 0.029 cm/s for 7 °, 25 ° and 35 °C, respectively. This corresponds to a temperature dependence of P_O2_ at 0% cholesterol of 6.5%/°C, whereas O_2_ diffusion in water exhibits a temperature dependence of about 3%/°C [17]. Figure 7 also shows the dependencies of P_O2_ on membrane cholesterol for the three temperatures. As already apparent in Figs. 3 and 4, the resistance of the membrane for O_2_ decreases, and its permeability increases, when the cholesterol fraction in the membrane is raised. This holds equally for all temperatures studied. However, between 0 and 20 mol% cholesterol, there is little effect of cholesterol on P_O2_. This implies that “low cholesterol” membranes (0–20%) exhibit the highest resistance toward the transfer of O_2_. Only when cholesterol increases from 30 to 70% (“high cholesterol”), there is a clear increase in permeability and thus a decrease of O_2_ diffusion resistance. This increase in P_O2_ amounts to about a factor of 10 between 0 and 70 mol% (a little less for the 60 mol% at 25 °C). It is noteworthy that for all temperatures measured the most pronounced increase in P_O2_ occurs between 30 and 40 mol% cholesterol. At 50% cholesterol and above, the kinetics of Hb-loaded liposomes approach quite closely the kinetics of the pure hemoglobin solution (Fig. 4). Interestingly, at these cholesterol fractions, the temperature dependence of P_O2_ is small or absent (Fig. 7).

## Discussion

### Limitations of the present experimental approach and comparison with the other approaches

The present stopped-flow technique derives O_2_ membrane permeability from the transmembrane O_2_ concentration gradient and from the flux of O_2_ across the liposome membrane. While the extraliposomal O_2_ partial pressure (pO_2_) is always nearly zero due to the presence of dithionite (see explanations in “Methods”, section “Stopped-flow experiments”), the intraliposomal pO_2_ and the efflux of O_2_ can be derived from the changes in intraliposomal oxygen saturation of hemoglobin, which is the major measured parameter. Knowledge of the thickness of the membrane is not directly necessary for determining membrane P_O2_, but as is apparent from Eqs. 3 and 4 given in the “Methods” section,* Theory*, knowledge of the surface-to-volume ratio a/v is required and this quantity is affected by the assumed thickness of the membrane. a and v are obtained from the experimentally determined diameter of the liposomes (Fig. 2), assuming a spherical geometry, which is roughly in accordance with the appearance of the liposomes in transmission electron microscopic images (Fig. 1). In conjunction with this, we assume a membrane thickness of 5 nm. Two alternative approaches to calculate P_O2_ from the experimental half-times of the stopped-flow records are employed, as discussed in “Methods”, Theory, after Eq. 4 and after Eq. 6. The resulting uncertainty in t_1/2_ of the calculated deoxygenation kinetics, however, is shown there not to exceed 3%, which we consider to be of minor importance.

A major limitation of the present method is the lack of information provided on the intra-membrane inhomogeneity. We must treat the membrane as a “black box” and determine only its overall permeability. However, it has been shown in much detail, e.g. by molecular dynamics simulations, that the lipid bilayer is highly inhomogeneous with respect to free energy, O_2_ solubility and diffusivity [12, 13, 18, 19]. Our data shed no light on these intra-membrane properties.

As discussed in detail below, the present results are greatly at variance with P_O2_ values of lipid bilayers reported from several ESR measurements [11] and MD simulations [12, 13, 18–20] by one to four orders of magnitude. In addition, we find that membrane cholesterol increases P_O2_, whereas with ESR [11] and MD simulations [20, 21], a moderate decrease in P_O2_ is observed. While our data provide no molecular explanation for these discrepancies, it is obvious that there is a fundamental difference between the present and the two other mentioned techniques: in MD calculations as well as in ESR, O_2_ and the membrane are in equilibrium and no net flux of O_2_ occurs. In the stopped-flow approach, an initial O_2_ gradient is set up and we follow its decay, from whose kinetics we determine P_O2_. Possibly, this is the key to the discrepancy, although the responsible mechanism is not understood as yet. It should be noted, however, that the present approach simulates the in vivo situation, where O_2_ gradients drive the net uptake of O_2_ from the lung into the blood as well as the net release of O_2_ from the blood to the O_2_-consuming tissues.

### Role of unstirred layers and multilamellar liposomes

The effect of unstirred layers on permeability measurements across lipid membranes have often led to controversial discussions. Therefore, we will discuss here the role of unstirred layers in the present experimental approach. The P_O2_ values calculated from the stopped-flow records could in principle be underestimated by the presence of unstirred layers on the external surface of the liposomes and by intraliposomal unstirred layers. We believe that unstirred layers in the present measurements do not affect P_O2_ for the following reasons:

1) The present vesicles are so small that extravesicular unstirred water layer thicknesses constitute no significant diffusion resistance [22, 23]. From the hydrodynamic treatment of Landau and Lifschitz [24] (p. 224), it follows that under conditions of turbulent flow, as it occurs in the mixing chamber of the stopped-flow apparatus, the thickness of the unstirred boundary layer δ is proportional to the length of the object ℓ, if all other conditions are identical. In stopped-flow experiments analogous to ours with human red cells at 37 °C, an unstirred layer thickness of 0.7 µm has been reported [15]. With ℓ = 8 µm for red cells and ℓ = 100 nm for the present liposomes, a δ for the present liposomes of 9 nm is predicted. The apparent permeability of such a water layer for O_2_ would be D_O2_/δ = 3 ∙ 10^–5^ cm^2^/s/9 ∙ 10^–7^ cm = 33 cm/s (where D_O2_ is the diffusion coefficient of O_2_ in water [17]). This O_2_ permeability of the unstirred layer is at least two orders of magnitude greater than the permeabilities of Fig. 7. Thus, it can have no effect of the calculated P_O2_. A further, and equally striking, argument against a role of an extravesicular unstirred layer is the high concentration of dithionite (50 mM) in the solution with which the liposome suspensions are mixed in the stopped-flow apparatus. As the consumption of O_2_ by dithionite occurs at an about equimolar ratio, we have a huge O_2_ uptake capacity in the extravesicular space, orders of magnitude greater than the O_2_ present in the system. Due to the high speed of this reaction, this will the ensure that all O_2_ leaving the liposome will be consumed practically immediately by dithionite [as explained in “Methods” (“Stopped-flow experiments”)], even if there existed a poorly stirred zone on the liposome surface. Such a poorly stirred layer will acquire the dithionite concentration of the surrounding solution by diffusion within < 1 ms, the dead time of the stopped-flow instrument: assuming a diffusion coefficient of 10^–6^ cm^2^/s for dithionite and using Crank’s equations and graphical solution (25], pp. 14–15, Fig. 2.3) one calculates that within 1 ms an unstirred layer even of 500 nm thickness will have assumed the dithionite concentration of the surrounding solution simply by diffusion without any convection. In addition, it may be noted that an only 9-nm-thick layer around the liposome with a dithionite concentration of 50 mM can absorb 3 times as much oxygen as is initially present inside the liposome. We conclude that due to both, the presence of dithionite and the marginal thickness of the unstirred layers, extravesicular unstirred layers cannot affect the experimental P_O2_ in the present experiments.

2) An alternative factor reducing the apparent P_O2_ could be an intravesicular unstirred layer, meaning that intravesicular diffusion processes would slow down the process of O_2_ release by the liposomes. To study this, we have simulated the process of O_2_ release ignoring or taking into account the intravesicular diffusion processes of O_2_ and hemoglobin with the equation systems described in “Methods”, “Theory of O_2_ release by liposomes and calculation of O_2_ permeabilities”. We compare there a theoretical model assuming a stirred intravesicular volume with one that takes into account intravesicular diffusion processes. Half-times of the deoxygenation kinetics differ between these models by < 1% for a broad range of P_O2_ values.

Thus, in all the cases and over the entire range of possible P_O2_ values, the intravesicular diffusion has no effect on the half-time of the kinetics of O_2_ release, because the diffusion times inside the small vesicles are extremely short. This conclusion holds for all the temperatures used in this study. An additional calculation shows that with P_O2_ = 0.1 cm/s, the influence of intravesicular diffusion becomes detectable at vesicle radii > > 100 nm only. Thus, with the present vesicle radii of 50 nm, intravesicular diffusion, or the intravesicular “unstirred layer”, does not influence the estimated P_O2_ values. In conclusion, the present measurements are affected neither by extravesicular nor by intravesicular unstirred layer effects.

3) As discussed in conjunction with the electron micrographs of Fig. 1, only 12% of the liposomes without cholesterol are more than unilamellar, usually with a second membrane within the primary liposome. In contrast, all cholesterol-containing liposomes are purely unilamellar. To which extent can the presence of a second membrane on the inside of the outer membrane affect the measured O_2_ permeability? Ignoring the surface areas and assuming identical properties of outer and inner membranes, the diffusion resistance of the “bilamellar” liposomes is given by 2/P_O2_, while that of the unilamellar vesicle is 1/P_O2_. Considering uni- and bilamellar liposomes as parallel resistances towards O_2_, we obtain an overall inverse resistance of 1/R_total_ = 0.12 ∙ P_O2_/2 + 0.88 ∙ P_O2_ = 0.94 ∙ P_O2_ cm/s. In other words, the presence of a small fraction of multilamellar liposomes affects the estimated P_O2_ by no more than 6%, i.e., the effect is almost negligible. Thus, the P_O2_- concentration curves of Fig. 7 will remain practically unaltered.

### Oxygen and carbon dioxide permeability in membranes

The present determinations of oxygen permeability, in contrast to earlier approaches, are based on direct measurements of O_2_ flux across a phospholipid bilayer. At a cholesterol concentration of ~ 45% as it occurs in several cell membranes including red cells, we find a P_O2_ of about 0.2 cm/s at 35 °C (Fig. 7). As will be shown below, this permeability is sufficiently great to guarantee a complete O_2_ equilibration of the red cell interior under conditions of rest, and a 95% complete O_2_ exchange even at the short capillary transit times in the lung as they may occur under heavy exercise [26]. Thus, this permeability is sufficient for pulmonary O_2_ exchange, and likely also for O_2_ release in most tissues, in which O_2_ fluxes are not as great as they are required across the red cell membrane.

**Fig. 8:**
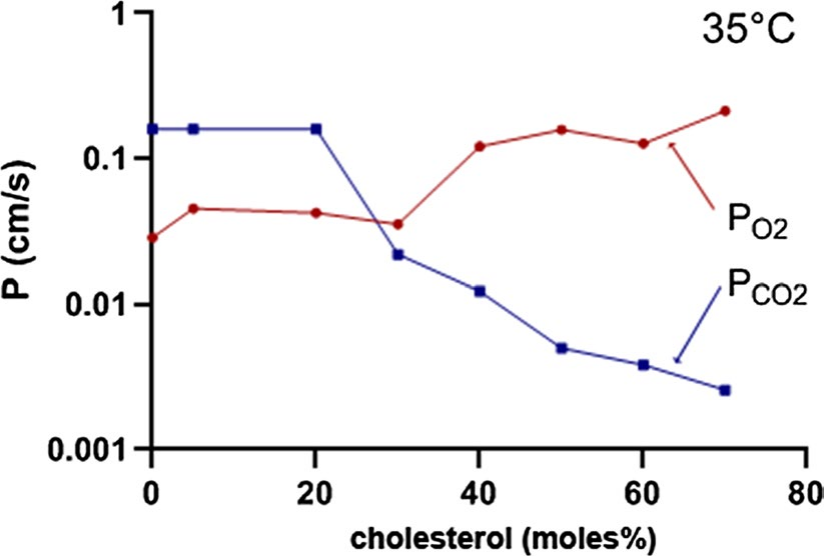
Permeability of phospholipid vesicles for O_2_ and CO_2_ in dependence on membrane cholesterol. P_O2_ represents the data of Fig. 7, P_CO2_ is from the mass spectrometric measurements of Itel et al. [2]

These results are remarkable in three important aspects that call for discussion:

1. The absolute value of P_O2_ determined here is much lower than has been derived from molecular dynamics simulations, which estimate P_O2_ values of 23 cm/s at 25 °C and 26–39 cm/s at 37 °C [12] or 20 cm/s at 25 °C [13]. This holds for the P_O2_ values of Fig. 7 with and without cholesterol. In addition, the present values are considerably lower than has been estimated from long-pulse saturation recovery ESR technique by the group of Subczynski [11], who report P_O2_ values of cholesterol-free phospholipid vesicles to be 125 cm/s at 29 °C. As discussed, unstirred layers on the external surface of the vesicles are not expected to play a role in our measurements and thus to reduce the apparent P_O2_. We note that Holland and coworkers [15], using a direct O_2_ flux measurement across the red cell membrane with a stopped-flow technique, after correction for an unstirred layer, obtained a P_O2_ of 0.6–0.8 cm/s for this membrane at 37 °C. This is in a range comparable to our value of 0.2 cm/s for liposomes containing 45% cholesterol.
  Oxygen permeability of liposomes vs. CO_2_ permeability: Figure 8 shows a comparison of the dependencies of P_O2_ and P_CO2_ of liposomes on membrane cholesterol. The former represent data from Fig. 7, the latter have been obtained by the mass spectrometric method applied to liposomes [2]. We note that these mass spectrometric results for P_CO2_ have qualitatively been confirmed by a stopped-flow technique [3]. While at the cholesterol concentration of the red cell membrane of 45% P_O2_ is 0.2 cm/s as just discussed, P_CO2_ is 0.008 cm/s, i.e., 25 times lower. Both curves refer to temperatures of 35 and 37 °C, respectively. This suggests a capability of membranes to become poorly permeable to CO_2_ by incorporating high concentrations of cholesterol [2, 3], a property that is physiologically useful in some apical endothelial membranes [27–29]. On the other hand, in membranes possessing high cholesterol levels such as red cells, a required high permeability to CO_2_ is achieved by the incorporation of protein CO_2_ channels aquaporin-1 and Rhesus-associated glycoprotein, which results in a P_CO2_ of the human red cell membrane of ~ 0.15 cm/s [9, 10]. Figures 7 and 8 show that membrane cholesterol does not constitute a problem for O_2_ transfer as P_O2_ is up to ten times higher than it is in the absence of cholesterol and also considerably higher than P_CO2_ above cholesterol concentrations of 30% (Fig. 8). We conclude that a membrane possessing a high cholesterol content in conjunction with protein CO_2_ channels exhibits high permeabilities for CO_2_ as well as for O_2_, plus offers other advantages of high cholesterol such as a general barrier function as explained below and enhanced mechanical stability of the membrane.
2. It is well known that membrane cholesterol constitutes a drastic barrier towards uncharged hydrophilic molecules such as water and NH_3_, but also formamide, acetamide, urea and glycerol [30], just as we show for CO_2_ in Fig. 8. Oxygen, a small but relatively hydrophobic and lipophilic molecule (see, e.g., solubilities as compiled in [31]), differs radically from this behavior as shown in Figs. 7 and 8. P_O2_ increases with increasing membrane cholesterol. This is a novel finding that will favor O_2_ transfer across high-cholesterol cell membranes, possibly without necessitating the presence of gas channels. As in the case of the absolute values of P_O2_ reported here, this finding is also in contrast to ESR-based measurements and molecular dynamics simulations. The ESR studies [11] predict a reduction of P_O2_ by 50% cholesterol to 1/5 of the value in the absence of cholesterol. Molecular dynamics simulations predict an only moderate reduction of O_2_ permeability of phospholipid bilayers in the presence of cholesterol by 20% [20, 21, 32]. At present, there is no explanation for the discrepancy to the measurements of O_2_ flux across the liposome membrane reported here. We note that increasing incorporation of the highly lipophilic cholesterol [33] into a phospholipid bilayer, besides condensing the membrane structure [34], will increase the amount of hydrophobic material present in the center of the membrane und may thus favor the passage of a small lipophilic molecules like O_2_ while increasing the hindrance to more hydrophilic molecules.

It is important to note that at all temperatures studied (Fig. 7), P_O2_ shows a step-like increase between 30 and 40 moles% cholesterol by almost one order of magnitude. This indicates that between these concentrations a major structural change occurs in the lipid bilayer that produces this tenfold increase in O_2_ permeability. While the structural cause of this abrupt increase is not clear, it is known from studies of the elastic properties of phospholipid bilayers that between 30 and 50 moles% cholesterol in the membrane a similarly abrupt decrease in the critical area strain and in membrane toughness occurs [35]. This clearly indicates a major structural change in this concentration region associated with a decrease in cohesive energy density of the membrane-constituting material.

In conclusion, the present results indicate that the O_2_ permeability of phospholipid membranes at high cholesterol, P_O2_ = 0.2 cm/s at 35 °C, are much lower than has previously been proposed. Second, P_O2_ depends inversely on membrane cholesterol compared to P_CO2_: P_O2_ increases with increasing cholesterol by about one order of magnitude, while P_CO2_ decreases with increasing cholesterol by up to two orders of magnitude. At a lipid composition of the membrane similar to that of human red cells, P_O2_ is 25 times higher than P_CO2_. This seems to make sense as a basic membrane property, as the gradients of dissolved O_2_ between extra- and intracellular space, due to the 20 times lower solubility of O_2_ compared to CO_2_, are considerably smaller than those for CO_2_.

### Physiological significance of oxygen permeability and possible role of O_2_ channels

**Fig. 9:**
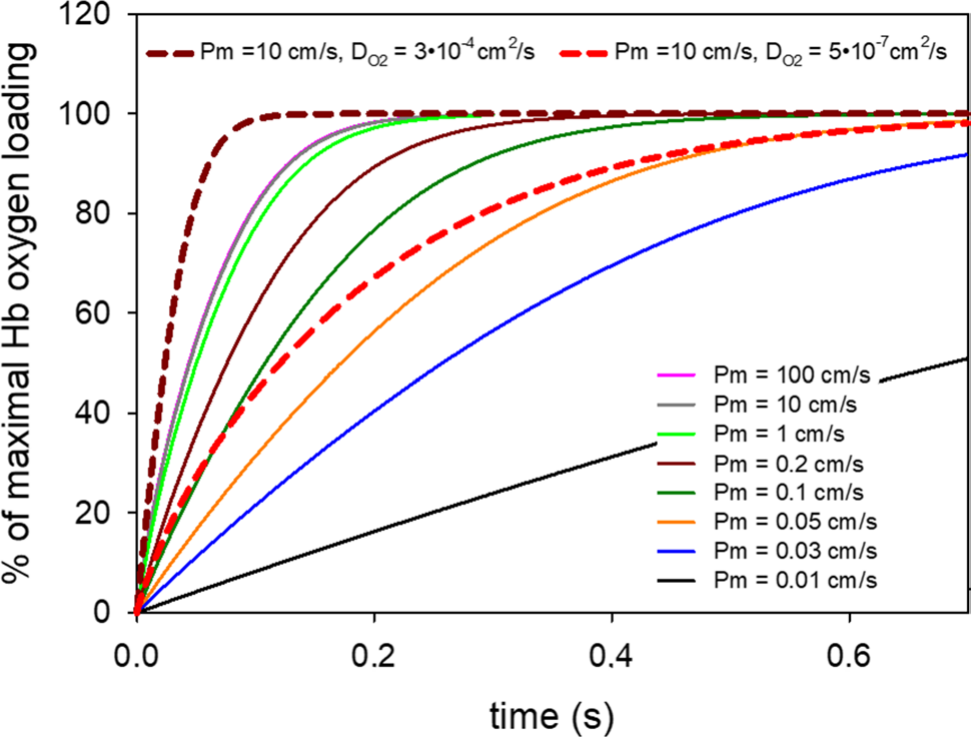
Calculated time course of red cell oxygenation during passage through the lung capillary. The dashed curves are calculated for extremely high or extremely low intraerythrocytic O_2_ diffusion coefficients together with an extremely high P_O2_ (see numbers at the top of the figure). The solid curves are calculated with an intraerythrocytic O_2_ diffusion coefficient of 0.8 ∙ 10^–5^ cm^2^/s and a wide range of O_2_ permeabilities from 0.01 to 100 cm/s. Note that the curves for 100 and 10 cm/s coincide completely (left-most continuous curve)

What do the numbers for P_O2_ found here mean in terms of oxygen exchange in the body? We will study this question for the example of O_2_ uptake by red cells in the lung. However, the answers will be similar for O_2_ transport in the tissue. Figure 9 shows the kinetics of O_2_ loading of red cells in the lung, calculated for different P_O2_ values of the red cell membrane. The equation system used is analogous to the one given in “Methods” for the release of O_2_ from spherical liposomes and considers chemical reaction and diffusion processes of Hb and O_2_ inside the red cell. However, the diffusion of O_2_ from the alveolus into the red cell was treated as a one-dimensional diffusion process in deviation from the use of spherical coordinates for the liposomes. Due to the parachute-like deformation the red cell experiences in the lung capillary [36], we used as the intracellular diffusion path the total average thickness of the red cell of 1.6 µm and added to this a 1-µm-thick unstirred water layer representing the thickness of the alveolar-capillary barrier. The latter assumption supposes that the membranes within the alveolar-capillary barrier constitute no significant diffusion barrier to O_2_. Figure 9 shows the results of these calculations for the physiological temperature of 37 °C. The major results are as follows: P_O2_ values of 100 and 10 cm/s coincide completely in the left-most continuous line, the line for P = 1 cm/s (bright green) deviates only very little from these two curves. In other words, physiologically it is irrelevant which P_O2_ value between 100 and 1 cm/s applies. If we consider the (purple) line for P_O2_ = 0.2 cm/s, i.e., the value we observe for a cholesterol concentration of 45% as it occurs in the red cell membrane (Figs. 7 and 8), we can conclude that with this P value a) we reach by far full equilibration of the red cell with the alveolar O_2_ partial pressure within the resting capillary transit time of 0.7 s, and b) even within the minimal capillary transit time seen under maximal exercise of 0.25 s [26], equilibration is still reached by about 95%. Thus, a P_O2_ of 0.2 cm/s guarantees a near complete equilibration under all physiological conditions. All curves for P_O2_ ≤ 0.05 cm/s do not even achieve full equilibration at a transit time of 0.7 s and by far not at 0.25 s. The dashed lines in Fig. 9 indicate the influence of intracellular diffusion of O_2_. The left-most dashed purple curve has been calculated for a D_O2_ of 3 ∙ 10^–4^ cm^2^/s, 38 times higher than the classical value of 0.8 ∙10^–5^ cm^2^/s for the red cell interior [37–39]. This is a condition in which the complete process is no longer limited by diffusion and the kinetics of the association reaction between Hb and O_2_ dominates. The dashed purple curve in comparison to the left-most continuous curve shows that roughly 1/2 of the normal red cell equilibration time is due to the limited speed of intracellular O_2_ diffusion. The second dashed curve (in red) has been calculated for an intraerythrocytic D_O2_ of 5 ∙ 10^–7^ cm^2^/s, which Richardson et al. [40] propose in analogy to their indirectly determined intraerythrocytic D_CO2_. This value is about 1/16 of the “classical” value of D_O2_ that has been used for the calculation of all the continuous curves in Fig. 9. The red dashed curve shows that such an extremely low intracellular O_2_ diffusivity would result in a severe deficit in oxygenation at a transit time of 0.25 s and would neither be quite sufficient for full equilibration after 0.7 s, even at an extremely high P_O2_ of 10 cm/s.

We should note that the red cell membrane is not just a cholesterol-containing lipid bilayer, but contains in addition at least 23% intrinsic protein [41]. Dotson and Pias [32] have reported that a similar protein content of the membrane reduces P_O2_ to about ½, supposing of course that this protein is O_2_-impermeable. This would lead us to a P_O2_ value for the red cell membrane of 0.1 cm/s (dark-green curve in Fig. 9). It is apparent that with such a P_O2_, O_2_ uptake in the lung would be complete after 0.7 s, but would reach no more than 80% of the maximum when only a transit time of 0.25 s is available. This would constitute a serious limitation of O_2_ uptake under conditions of heavy exercise. We know that no such limitation does in fact occur in healthy subjects. It may be speculated that here gas channels of the red cell membrane such as aquaporin-1 and Rhesus-associated glycoprotein might come into play. They are established as efficient CO_2_ channels in this membrane [9, 10], but molecular dynamics simulations have shown—at least in the case of aquaporin-1—that the CO_2_ channel can also act as an O_2_ channel [42]. This could well raise P_O2_ from 0.1 cm/s to 0.2 cm/s or even to the value of 0.6–0.8 cm/s proposed by Holland et al. [15], and thus guarantee a complete blood oxygenation in the lung even under conditions of heavy exercise.

## Methods

### Preparation of hemoglobin solutions

Red cell concentrate was obtained from the blood bank of the Medical School. Erythrocytes where washed 3 × in PBS at 2000 g for 10 min at 4 °C. The cell pellet was diluted 1:6 in distilled H_2_O and incubated for 1 h at 4 °C. To produce red cell membrane ghosts, the suspension was reconstituted to 150 mM NaCl and incubated for 30 min. Ghosts were then removed via centrifugation 2 times for 1 h at 16,000 g at 4 °C and the supernatant was decanted. The final supernatant was then concentrated in an Amicon system (Merck Amicon, Fisher Scientific, Germany) to a hemoglobin concentration of ~ 20 g%. For this, the solution was filled into the Amicon chamber equipped with a 10 kDa cutoff filter using a nitrogen pressure of 5 bar. The ultrafiltration was performed under gentle stirring at 4 °C. Hemoglobin concentration was determined with Drabkin’s solution.

### Preparation of hemoglobin-loaded liposomes

Liposomes were prepared by standard methods [2]. Briefly, phospholipids L‐α‐phosphatidylcholine (PC; chicken egg, mixture of different fatty acid chain lengths with C16 and C18 dominating; Avanti Polar Lipids, Alabaster, AL, USA; product number 840051) and L-α-phosphatidylethanolamine (PE; chicken egg, mixture of different fatty acid chain lengths with C16 and C18 dominating; Avanti Polar Lipids, Alabaster, AL, USA; product number 840021) and cholesterol (≥ 99%, Sigma Grade; Sigma‐Aldrich) dissolved in chloroform were mixed at the desired molar ratios (PC:PE 8:2 and variable amounts of cholesterol). The lipid mixture was dried to form a smooth lipid film on the inside of a small glass vial, followed by high‐vacuum drying for ≥ 3 h. The lipid film was hydrated to a concentration of 3 mg/ml in a phosphate buffer (83 mM NaCl, 40 mM Na_2_HPO_4_, 9.4 mM NaH_2_PO_4_) containing 10 g% hemoglobin (6.2 ∙ 10^–3^ M heme), and the detergent n‐octyl‐β‐d‐glucopyranoside (β‐OG; Avanti Polar Lipids, Alabaster, AL, USA) was added to a concentration of 4% (w/v). The resulting mixture was incubated with intermittent agitation for 1 h at room temperature, loaded into dialysis tubing (Spectra/Por; Spectrum Labs, Rancho Dominguez, CA, USA) with a molecular cutoff of 3500 Da and dialyzed against 200 volumes of phosphate buffer for 24 h at room temperature. Buffer was exchanged 3 times during this period. The mixture was then centrifuged at 10,000 g for 10 min and the resulting supernatant was extruded through track-etched filters of decreasing pore size, and in the final step, extruded 15 times through a 0.1 μm track-etched filter (Nucleopore; Whatman, Maidstone, UK). Extravesicular material was removed on a Superdex 200 column (GE Healthcare, Little Chalfont, UK) using the above phosphate buffer. Liposomes were stored at 4 °C.

### Characterization of liposomes

#### Transmission electron microscopy (TEM)

Liposomes were pelleted and resuspended in a tenfold volume of fixation buffer (150 mM HEPES, pH 7.35, containing 1.5% formaldehyde and 1.5% glutaraldehyde) for 30 min at RT and overnight at 4 °C. Liposomes were postfixed in 1% osmium tetroxide 2 h at RT and 4% uranyl acetate at 4 °C overnight. After dehydration in acetone, samples were embedded in EPON. 50-nm-thick sections were post-stained with 4% uranyl acetate and lead citrate [14] and observed in a Morgagni TEM (FEI), operated in the bright field mode. Images were recorded at 80 kV using a 2 K side mounted Veleta CCD camera, binned to 1 K. The number of unilamellar vs. bilamellar liposomes in liposomes without cholesterol was determined by inspection of a total of 793 liposomes. 12% were found to possess two rather than one membrane. Liposomes with cholesterol exhibited unilamellar liposomes only.

#### Determination of vesicle size by dynamic light scattering (DLS)

DLS studies were performed using a Viscotek 802 instrument (Viscotek Corporation) equipped with a single mode fiber optics and a 50 mW diode laser (*λ* = 832 nm) at 20 °C. The vesicle suspension was suitably diluted in a phosphate buffer as described above. Prior to dilution, the buffer was filtered through a syringe filter (Minisart^®^) with a pore size of 0.2 μm (Sartorius, Germany). The measurement yielded the average radius of the liposomes and their size distribution.

#### Cholesterol content of liposomal membranes

This was estimated from the ratio of cholesterol concentration vs. phosphatidylcholine concentration in the membrane. Cholesterol was determined using the Amplex Red Cholesterol Assay Kit (Invitrogen). Choline was determined with the Phosphatidylcholine Assay Kit (Sigma Aldrich). Fluorescence intensities were measured in a FLUOstar Optima microplate reader (BMG Labtech) and converted to molar concentrations using calibration curves. The experimental ratios of the two molar concentrations were compared with the ratios expected from the molar ratios of the two lipids in the original lipid mixture.

### Stopped-flow experiments

Stopped-flow experiments were carried out in a Hi-tech Scientific SF-61 DX2 double mixing stopped-flow system from TgK Scientific Limited (Bradford-on-Avon, United Kingdom; 20 ml cuvette volume, dead time 1 ms). Syringes and mixing chamber were kept at 7 °, 25 ° or 35 °C, respectively. The hemoglobin absorbance of vesicle suspensions oxygenated in air was measured at a wavelength exhibiting a large absorbance difference between oxy- and deoyhemoglobin (436 nm). In the case of hemoglobin solutions, a higher hemoglobin concentration was used compared to the average Hb concentration of the liposome suspension, and absorbance was, therefore, recorded at a wavelength somewhat less sensitive to the absolute specific absorbances of oxy- and deoxy-Hb as well as the difference between them (470 nm). While the intravesicular Hb concentration was ~ 6.2 mM, the average Hb concentration of vesicle suspensions was about 0.005 mM. Thus, the volume fraction of vesicles in the suspensions was of the order of 0.08 vols %. In contrast to the low Hb concentration of liposome suspensions, free hemoglobin solutions had a higher concentration of 0.077 mM, which served to prevent dissociation of Hb tetramers into dimers. One syringe of the stopped-flow apparatus contained either a suspension of Hb-loaded vesicles in the above phosphate buffer or a free Hb solution in the same buffer. In both cases, hemoglobin was fully oxygenated by equilibration in air. The other syringe contained a solution of 50 mM sodium dithionite with 40 mM Na_2_HPO_4_, 9.4 mM NaH_2_PO_4_ and 8 mM NaCl that had been prepared under anaerobic conditions and adjusted to pH 7.4, similar to the dithionite solutions used by previous investigators of the deoxygenation kinetics of hemoglobin or red cells [15, 16]. Upon mixing, all available dissolved extravesicular O_2_ is consumed by dithionite extremely rapidly within the dead time of the stopped-flow apparatus (ca. 1 ms) [43, 44]. We have shown this by supplementing the equation system given below by an equation describing the kinetics of O_2_ consumption by dithionite and using the kinetic constant reported by Creutz and Sutin [44]. The calculation shows in addition that the kinetics of hemoglobin deoxygenation observed after the stop is not noticeably affected by the speed of the dithionite reaction. In this latter phase after the stop, the O_2_ diffuses out of the vesicles, thereby crossing the liposome membranes, into the surrounding dithionite solution, which immediately also absorbs this oxygen. Thus, O_2_ partial pressure in the extravesicular space is kept zero at all times [15]. The kinetics of deoxygenation of the intravesicular hemoglobin was followed by recording the absorbance at the wavelength given above. The procedure was identical for free hemoglobin solutions, except for a different wavelength being used, as stated above, to limit the size of the absorbance signal. Using the software implemented in the stopped-flow apparatus, we usually were able to fit the records from experiments with free Hb to a first-order exponential with R^2^ > 0.999, while the experiments with liposomes were fitted with the same quality to a second-order exponential. These exponentials provided excellent empirical descriptions of the experimental records that could be used to determine the half-times of the kinetics as shown in Fig. 4. In the case of the hemoglobin solution, the fitted first-order exponential of the form Absorbance signal = $A{\text{e}}^{{\left( { - k_{obs} \cdot t} \right)}}$ directly provides the rate constant of oxyhemoglobin dissociation *k*_*d*_ as given by the value of *k*_*obs*_ in the exponent. A in this term is the initial value of the signal at *t* = 0.

### Theory of O_2_ release by liposomes and calculation of O_2_ permeabilities

The stopped-flow experiment starts with fully oxygenated hemoglobin (Hb) inside the liposomes (O_2_ partial pressure pO_2_ = that of air) and dithionite outside the vesicles, which maintains pO_2_ = 0 outside throughout the experiment. It ends when all Hb inside the vesicles is fully deoxygenated. The kinetics of this process is determined by (a) the kinetics of dissociation of Hb and O_2_ within the vesicle, and (b) the diffusion of O_2_ across the vesicle membrane as governed by the membrane O_2_ permeability P_O2_.

The kinetics of the reactions of oxygen and hemoglobin inside the vesicle are described by

1 $$\frac{{\partial \left[ {{\text{O}}_{2} } \right]_{i} }}{\partial t} = k_{d} \cdot \left[ {{\text{HbO}}_{2} } \right] - k_{a} \cdot \left[ {{\text{Hb}}} \right] \cdot \left[ {{\text{O}}_{2} } \right]_{i} ,{\text{and}}$$

2 $$\frac{{\partial \left[ {{\text{HbO}}_{2} } \right]}}{\partial t} = - k_{d} \cdot \left[ {{\text{HbO}}_{2} } \right] + k_{a} \cdot \left[ {{\text{Hb}}} \right] \cdot \left[ {{\text{O}}_{2} } \right]_{i} ,$$

where [O_2_]_*i*_ is the intraliposomal concentration of dissolved oxygen, and [HbO_2_] and [Hb] are the concentrations of oxygenated and deoxygenated Hb, respectively. *k*_*a*_ is he association kinetic constant, and *k*_*d*_ is the dissociation kinetic constant.

Only dissolved oxygen is able to diffuse across the vesicle membrane and the resulting change in intravesicular oxygen concentration is described by

3 $$\frac{{\partial \left[ {{\text{O}}_{2} } \right]_{i} }}{\partial t} = P_{{{\text{O}}2}} \cdot \frac{a}{v} \cdot \left( {\left[ {{\text{O}}_{2} } \right]_{e} - \left[ {{\text{O}}_{2} } \right]_{i} } \right),$$

where *P*_O2_ is the oxygen permeability of the membrane, a is the surface area of the liposomes and *v* is the intravesicular volume. [O_2_]_*e*_ is the extravesicular O_2_ concentration = 0.

The right hand sides of Eqs. 1 and 3 can be added to give Eq. 4 that describes the actual change of intravesicular O_2_ concentration per time $\frac{{\partial \left[ {{\text{O}}_{2} } \right]_{i} }}{\partial t}$:

4 $$\frac{{\partial \left[ {{\text{O}}_{2} } \right]_{i} }}{\partial t} = k_{d} \cdot \left[ {{\text{HbO}}_{2} } \right] - k_{a} \cdot \left[ {{\text{Hb}}} \right] \cdot \left[ {{\text{O}}_{2} } \right]_{i} + P_{{{\text{O}}2}} \cdot \frac{a}{v} \cdot \left( {\left[ {{\text{O}}_{2} } \right]_{e} - \left[ {{\text{O}}_{2} } \right]_{i} } \right)$$

Equations 2 and 4 were then integrated numerically using MATLAB 2020a. P_O2_ was varied until the calculated kinetics exhibited a half-time identical to that of the experimental stopped-flow kinetics as shown in Fig. 3. When the external radius of the liposome is 50 nm and the thickness of the membrane is assumed to be 5 nm, the radius of the intravesicular volume containing the hemoglobin is 45 nm. a is then defined as the external surface of a sphere of 45 nm diameter, *v* is defined as the intravesicular volume of such a sphere. The membrane of a finite thickness is then replaced in this model by a surface of the area a and the O_2_ permeability P_O2_. This simplified model requires short computation times, but a) ignores the finite thickness of the membrane, and b) assumes a stirred intravesicular volume with the absence of any concentration gradients. Nevertheless, as discussed below, it generates quite correct results, which deviate from those of a more realistic model only very slightly.

We have also used a more complex model, which a) considers the actual thickness of the membrane, and b) considers the simultaneous reaction and diffusion processes in the vesicle interior. For this purpose, the above equations were expanded to a more complex model as follows:

The chemical reactions were again described by Eqs. 1 and 2, and, following Crank’s [25] treatment, the diffusion of O_2_ and HbO_2_ inside the vesicle was described by

5 $$\frac{{\partial \left[ {{\text{O}}_{2} } \right]}}{\partial t} = {\text{D}}_{{{\text{O}}_{2} }} \cdot \frac{1}{r} \cdot \frac{\partial }{\partial r}\left( {r^{2} \cdot \frac{{\partial \left[ {{\text{O}}_{2} } \right]}}{\partial r}} \right) = {\text{D}}_{{{\text{O}}_{2} }} \cdot \left( {\frac{{\partial^{2} \left[ {{\text{O}}_{2} } \right]}}{{\partial r^{2} }} + \frac{2}{r} \cdot \frac{{\partial \left[ {{\text{O}}_{2} } \right]}}{\partial r}} \right)$$

6 $$\frac{{\partial \left[ {{\text{HbO}}_{2} } \right]}}{\partial t} = {\text{D}}_{{Hb_{{}} }} \cdot \left( {\frac{{\partial^{2} \left[ {{\text{HbO}}_{2} } \right]}}{{\partial r^{2} }} + \frac{2}{r} \cdot \frac{{\partial \left[ {{\text{HbO}}_{2} } \right]}}{\partial r}} \right)$$

Again, the right hand sides of Eqs. 1 and 5 as well as those of Eqs. 2 and 6 are added to describe the combined contributions of intravesicular reaction and diffusion. These equations were solved by Crank’s finite difference method employing MATLAB. The entire sphere of radius 50 nm is divided into 1-nm-thick segments, within which reaction occurs and between which diffusion occurs. The complete sphere includes a membrane of an assumed 5 nm thickness, i.e., 5 segments. Integration is performed over the complete radius of 50 nm. However, the five layers representing the membrane lack hemoglobin, and thus, Eqs. 1, 2 and 6 are omitted. The problem of the transition between the membrane and the water phase was handled as described by Crank [24] in Eq. 8.45 on p. 150. The five membrane layers are given a homogeneous membrane D_O2,M_ that replaces D_O2_ in the above equations and together with the 5 nm thickness defines membrane P_O2_ = D_O2,M_/5 nm. This D_O2,M_ is varied until it gives calculated half-times which agree with the experimental half-times of the stopped-flow records.

#### Sensitivity of calculation for thickness of the membrane

In terms of the membrane barrier, the assumed membrane thickness is irrelevant, because the lower thickness of a thinner membrane will be compensated by a lower D_O2,M_, giving an identical P_O2_. However, in the complex model the membrane thickness is relevant in terms of the intravesicular volume. We have tested this effect using a modification of the described calculation by assuming a 4-nm-thick membrane, which is represented by four 1-nm-thick layers (complemented by 46 layers representing the vesicle interior). Of course, the values D_O2,M_ in these four 1-nm-thick membrane layers then turn out to be only about 4/5 of those in the five membrane layers. Evidently, the intravesicular volume is somewhat larger in this modified model. However, effects of the five- vs. four-layer model on the calculations are small. Between P_O2_ values of 0.03 and 0.2 cm/s, the deviations in calculated t_1/2_ are between < 1 and 2%. From this, we conclude that an uncertainty in the actual membrane thickness and the associated alteration of intravesicular water volume have no substantial consequence for the calculated t_1/2_ and thus for the estimated P_O2_.

#### Role of intravesicular diffusion

To make an approximate comparison possible between the simple model of Eq. 4, which assumes a stirred intravesicular volume, and the complex model, which takes intravesicular diffusion into account, we use the following approach. The complex model is equipped with a 1-nm-thick membrane, i.e., one 1-nm-thick layer for the membrane and 49 layers for the vesicle interior. This geometrical situation comes close to the situation in the simple model of a vesicle of 50 nm radius, in which the membrane has in effect no thickness. Thus, a difference between the two modes of calculation can approximately be attributed to the effect of intravesicular diffusion. Between P_O2_ values of 0.03 and 0.2 cm/s, the deviations in calculated t_1/2_ are all ≤ 1%. This result is unaltered, when we use in the complex model a membrane thickness of 0.5 nm instead of 1 nm. This shows that intravesicular diffusion contributes entirely insignificantly to the deoxygenation kinetics of the liposomes.

Overall, the computations with the simple model provide a very good approximation of the result obtained with the complex model. This allowed us to first apply the experimental results to model one, and then use the result to obtain with little or no fitting the final result on the basis of the complex model with 5 membrane layers. Deviations between the t_1/2_ values from the two modes of computation were no greater than 1–3%.

#### Boundary conditions and constants

The initial oxygen﻿ partial pressure pO_2_ within the vesicle was that of air, about 150 mmHg, and the intravesicular hemoglobin initially was maximally loaded with O_2_. The extravesicular pO_2_ was 0 mmHg at all times. The time intervals used in the numerical integration of the complex model must be sufficiently small to ensure stable numerical integration; usually a time interval of 0.1 ns was used, which made these computations rather time-consuming. The constants employed in the calculations were, at the temperatures 7 °, 25 ° and 35 °C, for D_O2_: 1.1 ∙ 10^–5^, 1.8 ∙ 10^–5^ and 2.4 ∙ 10^–5^ cm^2^/s ([17], corrected for intravesicular Hb after [45]), for solubility α_O2_: 2.33 ∙ 10^–3^, 1.60 ∙ 10^–3^ and 1.41 ∙ 10^–3^ mmol/l/mmHg [46], for oxygen affinity p_50_ in the absence of 2,3-BPG: 1.70, 6.31 and 12.6 mmHg [16], for D_Hb_: 34 ∙ 10^–8^, 51 ∙ 10^–8^ and 64 ∙ 10^–8^ cm^2^/s ([47], corrected for temperatures), respectively. The equilibrium constant *K*_*d*_ describing the equilibrium of Hb, O_2_ and HbO_2_ was obtained as α_O2_ ∙ p_50_. The rate constants *k*_*d*_ describing the dissociation of O_2_ from HbO_2_ used in the calculations were obtained from the present stopped-flow measurements mixing oxyhemoglobin solutions with dithionite solutions and had the values 5.62, 56.8 and 175 s^−1^. The rate constants *k*_*a*_ of the association of Hb and O_2_ were obtained as the ratios of *k*_*d*_ over the equilibrium constant *K*_*d*_.

## Data Availability

All the original data are available upon request, and sources of material are given in the text.
